# Task shifting in health service delivery from a decision and policy makers’ perspective: a case of Uganda

**DOI:** 10.1186/s12960-018-0282-z

**Published:** 2018-05-02

**Authors:** Sebastian Olikira Baine, Arabat Kasangaki, Euzobia Margaret Mugisha Baine

**Affiliations:** 1 Department of Health Policy, Planning and Management, Makerere University College of Health Sciences, School of Public Health, P.O Box 7072, Kampala, Uganda; 2 Quality Assurance Directorate, Makerere University, P.O Box 7072, Kampala, Uganda

**Keywords:** Task shifting, Higher skilled health workers, Competences, Support supervision

## Abstract

**Background:**

Documented evidence shows that task shifting has been practiced in Uganda to bridge the gaps in the health workers’ numbers since 1918. The objectives of this study were to provide a synthesis of the available evidence on task shifting in Uganda; to establish levels of understanding, perceptions on task shifting and acceptability from the decision and policy makers’ perspective; and to provide recommendations on the implications of task shifting for the health of the population in Ugandan and human resource management policy.

**Methods:**

This was a qualitative study. Data collection involved review of published and unpublished literature, key informant interviews and group discussion for stakeholders in policy and decision making positions. Data was analyzed by thematic content analysis (ethical clearance number: SS 2444).

**Results:**

Task shifting was implemented with minimal compliance to the WHO recommendations and guidelines. Uganda does not have a national policy and guidelines on task shifting. Task shifting was unacceptable to majority of policy and decision makers mainly because less-skilled health workers were perceived to be incompetent due to cases of failed minor surgery, inappropriate medicine use, overwork, and inadequate support supervision.

**Conclusions:**

Task shifting has been implemented in Uganda for a long time without policy guidance and regulation. Policy makers were not in support of task shifting because it was perceived to put patients at risk of drug abuse, development of drug resistance, and surgical complications.

Evidence showed the presence of unemployed higher-skilled health workers in Uganda. They could not be absorbed into public service because of the low wage bill and lack of political commitment to do so.

Less-skilled health workers were remarked to be incompetent and already overworked; yet, the support supervision and continuous medical education systems were not well resourced and effective.

Hiring the existing unemployed higher-skilled health workers, fully implementing the human resource motivation and retention strategy, and enforcing the bonding policy for Government-sponsored graduates were recommended.

## Background

Task shifting has been practiced in many countries for years as a means to address the shortage of higher-skilled health workers [1–3]. Shortage of higher-skilled health workers is a global quandary [3–6]. It is worst in low- and middle-income economies (including Uganda). In Uganda, health staffing levels are as low as 50% or less of the established positions in hard-to-reach-and-stay rural areas [7–9].

The causes are complex and range from failure to train adequate numbers of skilled health workers, failure to attract and retain them, deaths, migration, to complex political-socio-economic contexts. Poor work conditions, benefit packages, and job satisfaction cause migration of health workers in search of better packages from other countries, the private sector within countries, and others exit the health sector [10–12].

Uganda introduced a bonding policy in an attempt to retain her employees (including health workers) after training. Government-sponsored students sign an agreement with Government to work for the Public Service for 3 years following completion of the training. Officers who sponsor themselves but are granted study leave are bonded for a period not exceeding the length of the training following completion of the course. Director of public prosecution has powers to recover from the officer costs of training in case of breach of the bonding agreement [13, 14]. However, enforcement of the bonding policy has been weak.

Shortage of higher-skilled health workers led to the birth and growth of task shifting which has been perceived as a magic bullet to address the health workforce shortage [15–18]. According to WHO, task shifting is defined as “a process of delegation whereby tasks are moved, where appropriate, to less-specialized health workers” [2, 3]. Documented evidence shows that task shifting in Uganda started in 1918 [19]. Task shifting was practiced at all levels of care including hospitals and more so in rural settings because there were no incentives to attract and retain higher-skilled health workers. The roles of higher-skilled health workers were assigned or delegated to less-skilled health workers, e.g., roles of doctors such as cataract extraction, medical male circumcision, surgical toilet, episiotomies, tooth extraction, and prescription of medicines were delegated to clinical officers or nurses and midwives [20].

Evidence shows increased access to and improved quality of care of HIV services in Brazil, Ethiopia, Malawi, Namibia, and Uganda when specific tasks were delegated to community health workers. Community health workers (CHWs) and people living with HIV/AIDS in Malawi and Uganda supported non-specialist doctors or nurses to deliver basic care package for people living with HIV/AIDS [21, 22]. Non-physician clinicians have been a cornerstone in the antiretroviral treatment strategies Democratic Republic of Congo, Ethiopia, Malawi, Mozambique, Tanzania, Uganda, and Zambia [3, 20, 23–28].

In Uganda, CHWs played a substantial role in integrated community case management programmes such as drug distributors in community-directed treatment interventions against onchocerciaisis, schistosomiasis, and intestinal helminth infections [29–32]; mobilizers and supervisors for maternal and child health, HIV/AIDS, tuberculosis, pneumonia, and malaria programs; and data collectors for audits [33].

Surgical tasks have been effectively performed by non-specialist physicians and non-physician clinicians with little supervisory or training framework in place in the absence of high-skilled surgeons [2, 3, 19]. However, the limits of task shifting must be precisely defined and regulatory bodies adequately engaged. Less-skilled health workers must receive adequate training, support supervision, continuous medical/professional education, recognition, remuneration, and simplified tools and guidelines to avoid compromising quality of care and putting the population at risk [34–38].

Although task shifting is perceived as means to respond to the shortage of higher-skilled health workers, there are several challenges to its implementation: lack of adequate and sustainable training, support supervision by competent health workers, motivation, work overloads, and non-compliance with the WHO guidelines and recommendations [39, 40].

### Statement of the problem

Documented evidence shows that task shifting has been practiced in Uganda as a means to address the shortage of higher-skilled health workers since 1918 [19]. Much has been talked, but little has been done to formulate a policy to support it. Substantive evidence shows that it has been informally implemented without appropriate standard national guidance. This raises questions about decision and policy makers’ perceptions, acceptability, and feasibility to implement task shifting in compliance with the WHO recommendations and guidelines.

### Objectives of the study

The objectives of this study were

- To provide a synthesis of the available evidence on task shifting in Uganda,
- To establish levels of understanding, perceptions on task shifting and acceptability from the decision and policy makers’ perspective, and
- To provide recommendations on the implications of task shifting for the health of the population in Ugandan and human resource management policy.

## Methods

This was a qualitative study by design aimed at gaining in-depth understanding of task shifting, implementation and effects (wins and losses) peculiar to Uganda. Participants at the national level included health policy and decision makers drawn from the Ministry of Health, Uganda Catholic Medical Bureau, Uganda Protestant Medical Bureau, Uganda Muslim Medical Bureau, World Health Organization Uganda Country office, Uganda National Health Consumers Organisation, Uganda Medical and Dental Practitioners Council, Uganda Dental Association, Uganda Nurses and Midwives Council, Uganda Allied Professional Council, and Capacity Program Uganda. Participants at the district included District Health Officer, in-charge of a health sub district, and the Resident District Commissioner.

Representatives of these institutions were purposively selected and priority was given to those in leadership and policy-making positions. The motivation was to generate rich information on task shifting and health policy-making process especially human resources for health. The District Health Officer and the In-Charge of a health subdistrict gave the implementers’ perspective. The Resident District Commissioner and Uganda National Health Consumers Organization (an advocacy Civil Society Organization for the people in need of health care) represented the consumers of health services.

Data were collected in a three-stage process. First, we reviewed published and gray literature in the Constitution of Uganda 1995, Local Government Act (1997), National Health Policy, Human Resource Policy, Motivation and Retention Strategy for Human Resource for Health, Annual Health Sector Performance Report 2014/15 and searches from electronic databases such as PubMed, Cochrane Library, and Social Science Citation Index.

Secondly, we interviewed 12 key informants holding senior positions in their institutions to gain clarification on particular issues relating to task shifting. Open-ended questions were used to interview participants from a wide and varied background on their day-to-day knowledge of and experiences with task shifting.

Thirdly, a group discussion of 16 representatives drawn from the Ministry of Health, Development Partners, Professional bodies, Civil Society Organizations/NGOs, and the academia was conducted. Group discussion participants were from different organizations and agencies. These individuals had not participated in key informant interviews because we wanted to avoid the Hawthorne effect and bias, and to observe dynamics and gain consensus on task shifting issues from different stakeholders with different perspectives.

During the interviews and group discussions handwritten notes were taken. The notes were used to fill gaps where recordings were not clear because either the voice reduced or background disruptions overshadowed the respondents’ words. Handwritten notes also helped to capture useful information from body language. The Principal Investigator stored and retained all data (raw and processed) throughout and after the study.

Co-Principal Investigators transcribed data verbatim, and the Principal Investigator confirmed the data after a series of listening to the recorded data and transcriptions. Data from the different sources were triangulated and coded, and themes were generated. The themes included shortage of health workers; policy and legal framework; recruitment; deployment and retention of high-skilled health workers; support supervision; training of health workers; community health workers; opinions about task shifting; World Health Organization guidelines and recommendations; acceptability of task shifting by policy and decision makers; and challenges faced in task shifting. A thematic content analysis was then conducted.

### Ethical considerations

The study received ethical approval from Makerere University School of Public Health Higher Degrees, Research and Ethics Committee and Uganda National Council of Science and Technology (SS 2444). Participants were adequately informed of the aims, methods, sources of funding, institutional affiliation of the researchers and the anticipated benefits. They were assured of no harm as a consequence of participating in this study or predictable risks and burdens. They were also informed of their right to exit the study or withdraw consent to participate at any time without reprisal. Informed consent was obtained from each participant prior to starting the key informant interviews, group discussions, and tape recording. Precautions were taken to protect the privacy of the subjects and confidentiality of personal information and to minimize the impact of the study on their physical, mental, and social integrity. Participants gave informed consent without duress. All forms of data stored and retained by the Principal Investigator throughout and after the study.

### Limitation to the study

Health providers at the implementation level and consumers of health services did not participate in this study, and their views on task shifting could have been different and enriching. A comprehensive study including them should be designed. The study had limited funding support.

Due to limited funding, participants from one district level were included. This meant that views from other districts that could enrich this study were missed out.

## Results

This section presents key findings of the study. The consensus was that a severe shortage of higher-skilled health workers existed in Uganda and it needed to be addressed with urgency. Policy and decision makers concurred that

The health sector faced an enormous shortage of higher skilled health workers. Filled established positions increased to about 68% after the mass recruitment of health workers in 2013.

Participants acknowledged that task shifting has been at the center of health service delivery in Uganda for a long time. Less-skilled health workers (clinical officers, nurses, midwives, laboratory assistants, microscopists, public dental health officers, anesthetic assistants) performed duties of skilled health workers. For example, nurses and midwives prescribed medicines and performed episiotomies which were the domain for a physician and obstetrician respectively. Participants revealed that

Clinical officers managed post abortion patients and conducted minor surgery. Nurses gave anaesthesia during surgery and carried out medical male circumcision.

Task shifting stressed the already overloaded less skilled health workers, compromised quality of health care, and there was minimal compliance to WHO guidelines and recommendations.

### Task shifting policy and legal framework

Uganda has no task shifting policy and legal framework to guide the implementation of task shifting. Task shifting was informally implemented and not regulated, and less-skilled health workers risked prosecution in courts of law in case of causing death or disability in the process of performing delegated roles. Participants from the Ministry of Health revealed that

The task shifting policy had not been formulated because it would be technically difficult to cancel it once enacted basing on past experience in Uganda and the process involved.

### WHO guidelines and recommendations

The WHO guidelines and recommendations were developed to guide countries that implemented task shifting. Requirements entailed a significant amount of resources that were not readily available in the health sector. All participants concurred that

It was more expensive to implement task shifting as recommended by WHO than recruiting the already higher skilled health workers who were unemployed.

### Recruitment, deployment, and retention of higher-skilled health workers

The Health Service Commission, on behalf of the Ministry of Public Service and District Service Commission, were mandated to recruit health workers at the national and district levels respectively. Participants reported the presence of many unemployed doctors due to the Government ban on recruitment of new human resources in general, and the small wage bill could not allow employment of unemployed higher-skilled health workers. Participants from the Ministry of Health revealed that

Higher skilled healthcare workers were actually available but could not be hired because of the low wage bill and government veto on recruitment.

Many higher skilled Ugandan health workers migrated due to lack of a clear career development path, poor work conditions and benefit packages.

Health managers, civil society organizations, and decision makers held an opinion that Government did not attach significant value to higher-skilled health workers. This was premised on the existence of unemployed higher-skilled health workers, poor working conditions, and remuneration packages not matching workload. Against this backdrop, participants reached a consensus that

There were higher skilled health workers who were not employed. Government should first deploy the existing higher skilled health workers before investing in task shifting.

### Support supervision

Less-skilled health workers taking on more responsibilities had not received adequate support supervision because existing competent health workers were few in number, overloaded with work, and lacked adequate resources and incentives. Support supervision was inadequate and erratic, and quality assurance was not guaranteed. Participants argued that less-skilled health workers put patients at risk while one participant from an NGO asserted that they were safe if well mentored and supervised by competent health workers.

### Training of health workers

Training curricula for less-skilled health workers was limited in content and did not empower them to perform a wide range of health interventions. Continuing medical education and support supervision were mandatory to ensure competency improvement. However, continuing medical education was not well streamlined into health services and did not necessary empower health workers to take on new responsibilities. Participants argued that

Continuing medical education was not a motivator because they put in a lot of effort and time to become more competent but there were no investment returns in terms of career progression, incentives, benefits package or salary increment.

### WHO guidelines and recommendations

Participants argued that it was expensive for Government to implement task shifting following WHO guidelines and recommendations. Resources are scarce, and implementation of WHO guidelines and recommendations was not feasible. Recruitment from the available higher-skilled health workers that were unemployed was perceived cheaper to implement than task shifting according to WHO guidelines and recommendations [12].

### Opinions about task shifting

Consumers of health services were not informed about task shifting and competences of different health workers and could not make informed decisions on the choice of health provider. All participants reported that

Patients preferred doctors to less skilled health workers. They became dissatisfied with health services on learning that they were not treated by doctors.

All participants held an opinion that competence and complexity of the procedure should be given due consideration prior to allowing less-skilled health workers to carry out surgery. For example, with targeted training and support supervision, clinical officers, midwives, and nurses could be allowed to do simple surgical procedures such as surgical toilet, episiotomy, and incision and drainage of an abscess..

Participants questioned why task shifting was practiced when there were many unemployed higher-skilled health workers in the country. Decision and policy makers acknowledged presence of unemployed higher-skilled health workers, a small wage bill, and a Government veto on recruitment as causes of shortage of higher-skilled health workers. Based on past experience, they sounded fearful on the difficultly to revoke a task shifting policy in the future once it becomes operational. Participants from the private sector expressed frustration because task shifting had been discussed in different forums but there was no policy and legal framework to guide its implementation.

A participant from the NGO sector perceived task shifting as an acceptable and cost-effective solution to health workforce shortages. An experience of how task shifting was applied to address the health workers shortage was given.

We trained and mentored nursing aides in our mission hospital. We provided close support supervision and they were able to setup intravenous drips.

### Acceptability of task shifting by policy and decision makers

There was no pronouncement by policy and decision makers in support of task shifting. It was not considered to be a solution to the shortage of high-skilled health workers. Less-skilled health workers were perceived to be incompetent to provide certain services and were already overworked, and most undocumented evidence indicated that they did not provide quality of care. Policy and decision makers concurred that

No patients desired to be cared for by less skilled health workers. Patients wanted doctors/physicians or surgeons with higher skills and competences to attend to them.

### Challenges faced in task shifting

Challenges faced in task shifting included

- Identification of less-skilled health workers who could take on additional responsibilities from higher-skilled health workers,
- Scarcity of resources to facilitate mentorship, in-service training, and support supervision,
- Higher-skilled health workers off-loading a lot of their responsibilities to less-skilled health workers at the cost of quality and safety of care,
- Resistance of higher-skilled health workers and decision and policy makers to task shifting was based on the premise that certain procedures could not be shifted to less-skilled health workers since they were not well-trained to offer them unsupervised.
- Lack of a national policy and frameworks to guide implementation of task shifting and protection of less-skilled health workers against legal actions in courts of law in case of unintentional errors.

## Discussion

Documented evidence shows that task shifting has been implemented in Uganda’s health system as a coping mechanism to fill gaps caused by shortages of higher-skilled health workers since 1918 [19]. Less-skilled health workers were prescribing medicines, conducting minor surgery, and providing preventive health services [24–27]. The scope of work for the less-skilled health workers was not precisely defined and tasks delegated were dependent on the discretion of higher-skilled health workers. There was no national policy and regulatory frameworks to guide implementation of task shifting and less-skilled health workers were at risk of prosecution in courts of law. Regulatory bodies were not adequately engaged in the task shifting practice.

Compliance to the WHO guidelines and recommendations for task shifting was minimal due to a lack of resources required to prepare less-skilled health workers to perform the role of well-trained health workers. Recruitment of unemployed higher-skilled health workers was perceived to be cheaper than task shifting if employers of less-skilled health workers adhere to steps provided in the WHO recommendations and guidelines [2, 13].

Support supervision for the less-skilled health workers was inadequate and erratic. Continuous medical education was just a requirement for the renewal of license to practice. There was no motivation to undergo further training because investment returns were invisible.

Lack of political commitment and adequate resource allocations, drugs, equipment, and support supervision caused redundancy and migration of health workers. There was no compensation for additional workload to health workers, and yet this was a critical incentive for successful implementation of task shifting. The push factors for migration, job satisfiers, and retention factors for health workers were difficult to address in the existing socio-economic context [10–12]. Participants supporting continuation of task shifting argued that

- Less-skilled health workers already provided care beyond the scope of their training giving the example of nursing assistants who set up intravenous drips on patient after mentorship and support supervision [29–33].
- Less-skilled health workers simply needed capacity strengthening through targeted training and support supervision to increase competences and quality of care.
- A national policy and regulatory frameworks were needed to guide the implementation of task shifting and protect the less-skilled health workers taking on new roles against legal implications.

Participants not supporting continuation of task shifting argued that

The WHO recommendations and guidelines on task shifting were biased to preventive services. They emphasized ensuring safety, effectiveness, efficiency, equity, and sustainable support supervision. These were awfully challenging to guarantee in a developing economy like Uganda.

Already, there were higher-skilled health workers who were not employed in Uganda. It was cheaper to hire them than to conform to WHO task shifting guidelines and recommendations was deemed to be more expensive. It was more feasible for Government to first employ the unwaged higher-skilled health workers; offer them competitive work benefit packages to discourage migration; and implement fully the motivation and retention strategy for human resource for health.

Less-skilled health workers were neither adequately trained nor competent to provide any form of care safely, efficiently, and effectively. They were already overloaded, and assigning them additional roles would compromise quality of care even more and put the population at risk.

The population is ignorant about the competences of health providers and health rights; otherwise, patients would frequently sue less-competent health workers over mismanagement and the compensation costs would be high.

## Conclusions

Task shifting in health service delivery has been informally practiced in Uganda for decades despite the existence of many unemployed higher-skilled health workers. It has been implemented more in preventive health service delivery than curative (clinical medicine and surgery) services. The scopes of work for the less-skilled health workers were not defined, and regulatory bodies were not adequately engaged.

Less-skilled health workers did not receive adequate pre-service training, support supervision, continuous medical/professional education, recognition, remuneration, simplified tools, and guidelines. This put the population at risk of inappropriate diagnosis and medication, development of drug resistance, and surgical complications.

There were no national policy and frameworks to underpin its implementation. Government had no intention to make a policy to guide less-skilled health workers taking on new responsibilities.

Pre-service curricula for the less-skilled health workers were not comprehensive. Continuous medical education sessions were not comprehensive, targeted, regular, and self-sponsored. Support supervision was erratic, not guaranteed, and not effective. Continuous capacity building and competency strengthening were therefore farfetched.

Uganda had many courses of action to increase the availability of higher-skilled health workers. These included

- Government to central recruitment of high-skilled health workers because hard-to-reach-and-stay districts did not have capacity to attract and retain them.
- Government to strengthen the enforcement of the bonding policy [13, 14]. Academic transcripts and certificates from the training institution, and registering with respective health professional bodies may be retained until the agreed time has been served after completion of the training
- Government should mobilize resources and implement the motivation and retention strategy for human resources for health fully to reverse push factors for migration, strengthen job satisfiers, and increase attraction and retention.
- Government should create a supportive work environment and ensure availability of equipment and medicines and amenities to diminish redundancy among health workers and risk migration in search for better work conditions.
- Training curricula should be reviewed regularly to ensure more competences and capacity to take on a wide scope of roles and enforce quality assurance and support supervision. Government should adequately resource the Ministry of Health and Ministry of Education and Sports.

## Abbreviations

AIDS: Acquired immune deficiency syndrome
CHWs: Community health workers
HIV: Human immune virus
WHO: World Health Organization
